# Identifying the integrated neural networks involved in capsaicin-induced pain using fMRI in awake TRPV1 knockout and wild-type rats

**DOI:** 10.3389/fnsys.2015.00015

**Published:** 2015-02-19

**Authors:** Jason R. Yee, William Kenkel, John C. Caccaviello, Kevin Gamber, Phil Simmons, Mark Nedelman, Praveen Kulkarni, Craig F. Ferris

**Affiliations:** ^1^Center for Translational NeuroImaging, Department of Psychology, Northeastern UniversityBoston, MA, USA; ^2^SAGE LabsSt Louis, MO, USA; ^3^Ekam ImagingBoston, MA, USA

**Keywords:** capsaicin, TRPV1, fMRI BOLD, fMRI methods, habenula, Papez circuit, pain networks

## Abstract

In the present study, we used functional MRI in awake rats to investigate the pain response that accompanies intradermal injection of capsaicin into the hindpaw. To this end, we used BOLD imaging together with a 3D segmented, annotated rat atlas and computational analysis to identify the integrated neural circuits involved in capsaicin-induced pain. The specificity of the pain response to capsaicin was tested in a transgenic model that contains a biallelic deletion of the gene encoding for the transient receptor potential cation channel subfamily V member 1 (TRPV1). Capsaicin is an exogenous ligand for the TRPV1 receptor, and in wild-type rats, activated the putative pain neural circuit. In addition, capsaicin-treated wild-type rats exhibited activation in brain regions comprising the Papez circuit and habenular system, systems that play important roles in the integration of emotional information, and learning and memory of aversive information, respectively. As expected, capsaicin administration to TRPV1-KO rats failed to elicit the robust BOLD activation pattern observed in wild-type controls. However, the intradermal injection of formalin elicited a significant activation of the putative pain pathway as represented by such areas as the anterior cingulate, somatosensory cortex, parabrachial nucleus, and periaqueductal gray. Notably, comparison of neural responses to capsaicin in wild-type vs. knock-out rats uncovered evidence that capsaicin may function in an antinociceptive capacity independent of TRPV1 signaling. Our data suggest that neuroimaging of pain in awake, conscious animals has the potential to inform the neurobiological basis of full and integrated perceptions of pain.

## Introduction

The transient receptor potential vanilloid 1 (TRPV1) channel is a polymodal receptor critical to the sensing of noxious heat (>43°C), bidirectional changes in pH, and capsaicin, amongst a variety of other stimuli (Szallasi et al., 2007). Capsaicin, a pharmacologically active agent found in chili peppers, causes burning and itching sensation due to binding at the TRPV1 receptor, and subsequent activation of polymodal C and A-delta nociceptive fibers (Caterina et al., 1997, 2000; Tominaga et al., 1998). Acutely, TRPV1 activation with peripheral capsaicin produces pronociceptive effects, which extends to the development of hyperalgesia and allodynia (Simone et al., 1989; LaMotte et al., 1992; Torebjork et al., 1992; Ji et al., 2002). However, capsaicin has been reported to display antinociceptive properties as well, largely through TRPV1-dependent mechanisms (Szallasi and Blumberg, 1999; Palazzo et al., 2002; Starowicz et al., 2007; Chiou et al., 2013). Despite the promise for pain relief, mechanisms responsible for capsaicin's pro- vs. anti-nociceptive properties remain poorly understood (Brederson et al., 2013). The current study seeks to elucidate the neural response to peripherally administered capsaicin by employing functional magnetic resonance imaging (fMRI) in awake rats lacking TRPV1 receptors.

With functional imaging the neurobiology of capsaicin-induced pain in healthy volunteers has extended beyond primary sensory fibers to include distributed neural circuits (Iadarola et al., 1998; Baron et al., 1999; Maihofner and Handwerker, 2005; Zambreanu et al., 2005). The use of 3D segmented, annotated, brain atlases and computational analysis permits reconstruction of distributed, integrated neural circuits, or “finger prints” of brain activity (Ferris et al., 2011). Functional MRI at ultra-high magnet fields (≥7 Tesla) permits the study of brain activity across distributed, integrated neural circuits with high temporal and spatial resolution. Furthermore, the combination of awake fMRI with an imaging genetics approach (Hariri et al., 2006) represents a powerful experimental strategy that permits the identification of neural circuits regulating receptor-specific pain responses. While imaging genetics in humans takes advantage of natural polymorphisms to examine the genetic basis of differences in neural activation, the imaging of transgenic animals represents a more targeted approach that offers the potential to investigate the contribution of a single gene to neural response patterns. To our knowledge, these approaches have not yet been applied to the study of capsaicin-induced pain and pain relief.

To date there are only four published brain imaging studies on subcutaneous capsaicin-induced pain in rat and all have been under isoflurane or α-chloralose anesthesia (Malisza and Docherty, 2001; Malisza et al., 2003; Moylan Governo et al., 2006; Asanuma et al., 2008). Imaging the neural response to painful stimuli in awake animals allows identification of multiple, integrated neural systems involved in sensory discrimination, perception, motivation, and cognition. Borsook and Becerra (2011) reviewed the many benefits and applications for pain imaging in awake animals and the limitations introduced by anesthetics (Borsook and Becerra, 2011).

Here we report a series of neuroimaging experiments on a newly developed transgenic rat containing a biallelic deletion of the TRPV1 gene. We demonstrate proof of concept that TRPV1 is necessary for the production of an integrated neural response to capsaicin. We show data that inflicting pain in an awake animal during image acquisition not only activates pain neural circuitry but also neural circuitry involved in emotion and memory. In addition, our results suggest an antinociceptive role for capsaicin that is independent of TRPV1 signaling.

## Experimental methods

Adult wild-type, male Sprague–Dawley rats (*n* = 36) weighing ca 320–350 g were purchased from Charles River Laboratory (Wilmington, MA). Sprague–Dawley TRPV1 KO rats (*n* = 12) were provided by SAGE Laboratories (St. Louis, MO). Rats were maintained on a 12:12 h light:dark cycle with a lights on at 07:00 h. Animals were allowed access to food and water *ad libitum*. All rats were acquired and cared for in accordance with the guidelines published in the Guide for the Care and Use of Laboratory Animals (National Institutes of Health Publications No. 85–23, Revised 1985) and adhered to the National Institutes of Health and the American Association for Laboratory Animal Science guidelines. The protocols used in this study were in compliance with the regulations of the Institutional Animal Care and Use Committee at the Northeastern University.

### Awake animal imaging

#### Acclimation

A week prior to the first imaging session, all animals were acclimated to the imaging system before scanning. Animals were secured into their holding system while anesthetized with 2–3% isoflurane. Following cessation of isoflurane, fully conscious rats were put into a “mock scanner” (a black box with a tape recording of MRI pulses) for 30 min. Acclimation significantly reduces physiological effects of the autonomic nervous system including heart rate, respiration, corticosteroid levels, and motor movements (King et al., 2005) helping to improve contrast- to-noise and image quality.

#### Drug administration

Capsaicin (1 mg/ml) (Material # M2028 Sigma-Aldrich, St Louis, MO) was given subdermal in the right hindpaw in a volume of 50 μl via a 27-gauge needle connected to polyethylene (PE) 20 tubing extending from the bore of the magnet to a calibrated syringe. The same volume in the same hindpaw was given for formalin (3%) or 20% Captisol® (a modified β-cyclodextrin) vehicle (Ligand Pharmaceuticals, Inc. La Jolla, CA). Injections were separated by 2 weeks. All injections were performed in the magnet, under awake conditions during image acquisition. These doses are sufficient to trigger a pain response, yet small enough to avoid noticeable tissue damage.

#### Animal preparation with imaging protocol

Animals were scanned at 300 MHz using quadrature transmit/receive volume coil built into the rat head holder and restraining system for awake animal imaging (Animal Imaging Research, Holden, MA). The design of the coil provided complete coverage of the brain from olfactory bulbs to brain stem with excellent B1 field homogeneity. The design of the restraining imaging system included a padded head support obviating the need for ear bars helping to reduce animal discomfort while minimizing motion artifact. Imaging awake animals removes the obvious confound of anesthesia on activity of pain neural circuitry (Borsook and Becerra, 2011).

#### Image acquisition

Experiments were conducted using a Bruker Biospec 7.0T/20-cm USR horizontal magnet (Bruker, Billerica, Massachusetts) and a 20-G/cm magnetic field gradient insert (*ID* = 12 cm) capable of a 120-μs rise time (Bruker). At the beginning of each imaging session, a high-resolution anatomical data set was collected using a single-shot RARE pulse sequence (22 slice; 1.0 mm; field of vision [FOV] 3.0 cm; 256 × 256; repetition time [TR] 2.5 s; echo time [TE] 12 ms; NEX 2; 2 min acquisition time). Functional images were acquired using a multi-slice HASTE pulse sequence (*H*alf *F*ourier *A*cquisition *S*ingle *S*hot *T*urbo *S*pin *E*cho). A single scanning session acquired 22 slices, 1.0 mm thick, every 6.0 s (FOV 3.0 cm, matrix size 96 × 96, NEX 1) repeated 150 times for a total time of 15 min. Each scanning session was continuous, starting with 50 baseline image acquisitions, then hindpaw injection, followed by another 100 image acquisitions.

#### Data analysis

Data is co-registered to a mean functional image using SPM8's coregistrational code with the following parameters: Quality: 0.97, Smoothing: 0.35 mm, Separation: 0.5 mm. Gaussian smoothing was performed with a FWHM of 0.8 mm. Preprocessed functional files were then exported to Medical Image Visualization and Analysis (MIVA) for registration and segmentation. Images were aligned and registered to a 3D rat brain atlas, which is segmented and labeled with 137 discrete anatomical regions (Kulkarni et al., 2012). The alignment process was facilitated by an interactive graphic user interface. The affine registration process involved translation, rotation and scaling independently and in all three dimensions. Matrices that transformed each subject's anatomy were used to embed each slice within the atlas. All pixel locations of anatomy that were transformed were tagged with regions of interest in the atlas. This combination created a fully segmented representation of each subject within the atlas. The inverse transformation matrix [Ti]-1 for each subject (i) was also calculated.

In voxel-based analysis, the BOLD % change of each independent voxel was averaged for all subjects with a baseline threshold of 2% BOLD change to account for normal fluctuation of BOLD signal in the rat brain (Brevard et al., 2003). A composite image of the whole brain representing the average of all subjects was constructed for each group for ROI analyses, allowing us to look at each ROI separately to determine the BOLD change and the number of activated voxels in each ROI. Further details on the generation of composite activational maps are provided in Supplementary Materials. We also compared different points along the time course of the scan to compare the effects of capsaicin over time. For our analyses of the pain pathway, we looked at activation between 3 and 5 min post-capsaicin. We also compared activation at 2, 5, and 10 min, to compare activation of the stimulus over time.

**Figure 1:**
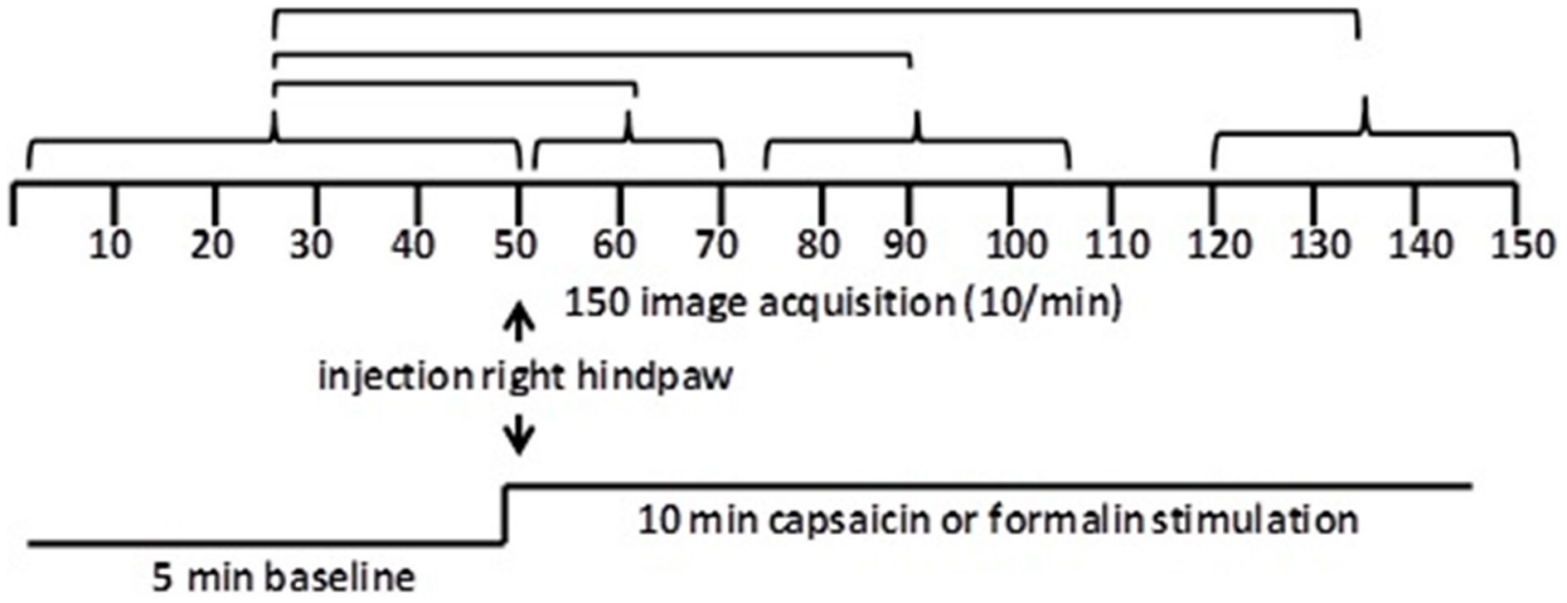
**Data analysis**. Shown are the statistical comparisons of different image acquisitions compared to baseline. A non-parametric Kruskal–Wallis test statistic was used to compare the average signal intensity in each of ca 15,000 voxel for their first 5 min baseline (acquisitions 1–50) to min 1–2 (acquisitions 51–71), min 3–5 (acquisitions 75–105), and min 8–10 (acquisitions 121–150) post-capsaicin and formalin.

The template shown in Figure 1 used to define the neural circuit of pain in the rat was derived from the work of Gauriau and Bernard (2002) and meta-analyses from various neuroimaging modalities used to study acute pain in humans (Apkarian et al., 2005). Using the parabrachial nucleus as a central node, Gauriau and Bernard described its many efferent connections to brainstem, hypothalamus, and forebrain areas as comprising the distributed neural circuit of pain (Gauriau and Bernard, 2002). They and others (Cechetto et al., 1985; Herbert et al., 1990; Bernard et al., 1994; Bourgeais et al., 2001b) have provided clear anatomical and electrophysiological evidence showing two major pathways of nociceptive fibers emanating from lamina 1 of the dorsal horn of the spinal cord.

#### Statistical analysis

**Figure 2:**
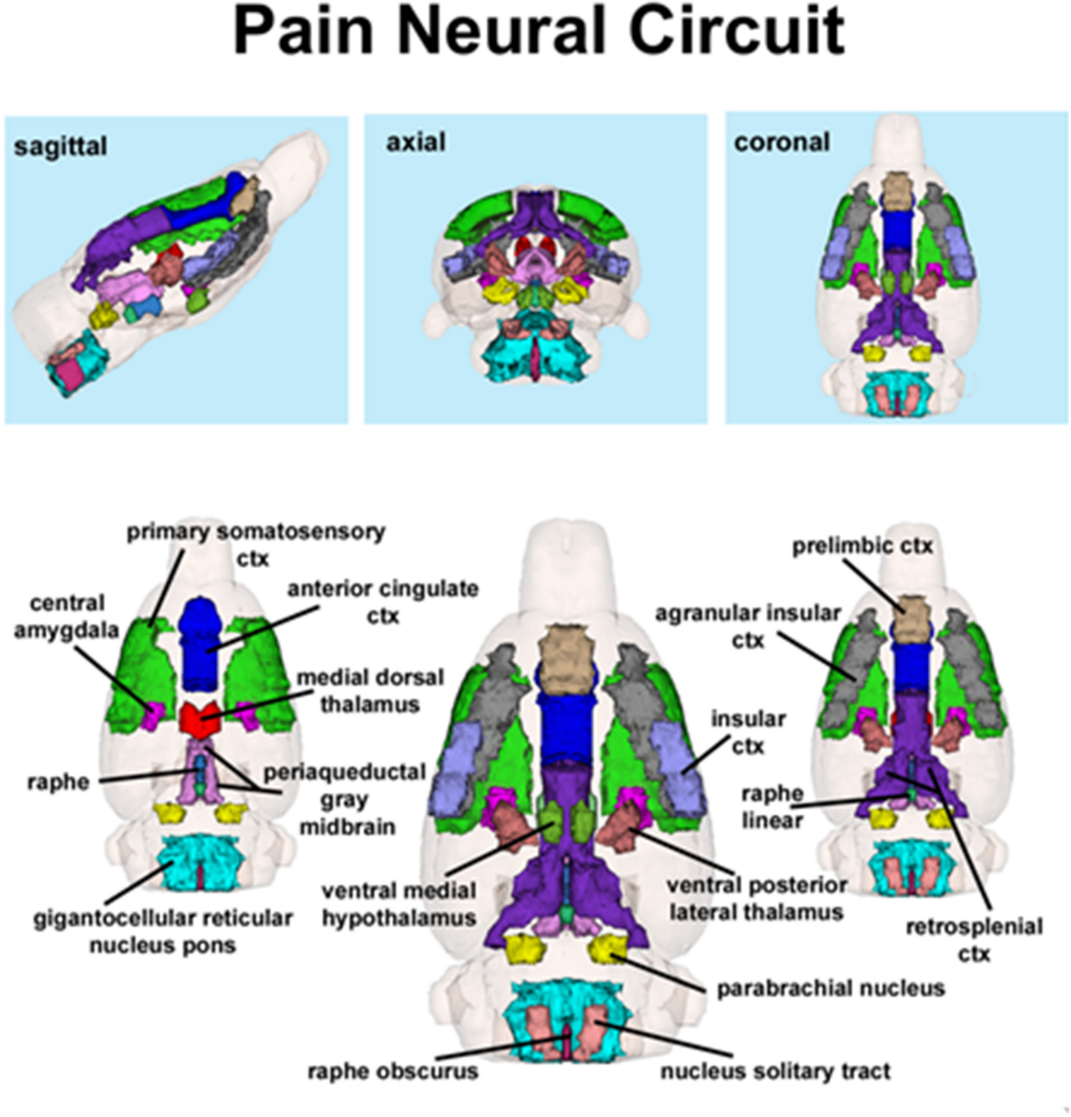
**Putative pain neural circuit**. Shown is a 3D representation of the different volumes that comprise the putative pain neural circuit. The central image is a coronal view of a translucent shell of the brain showing the total composite and location of the different 3D volumes. Surrounding this are different layers of the neural circuit showing a ventral (deepest) to dorsal perspective of the different brain volumes. The panels on the top show the neural circuit in different orthogonal directions. The template used to define the neural circuit of pain in the rat was derived from the work of Gauriau and Bernard (2002) and meta-analyses from various neuroimaging modalities used to study acute pain in humans (Apkarian et al., 2005).

Scanning sessions consisted of 150 data acquisitions each, with a period of 6 s for each image, for a total time lapse of 900 s or 15 min. Statistical *t*-tests were performed on each voxel (ca. 15,000 in number) of each subject within their original coordinate system. The average signal intensity in each voxel of the first 5 min of baseline (acquisitions 1–50) was compared to min 0–2 (acquisitions 51–71), min 3–5 (acquisitions 75–105), and min 8–10 (acquisitions 121–150) following administration of the pain stimulus or vehicle injection (Figure 2). The baseline threshold was set at 2%. The *t*-test statistics used a 95% confidence level, two-tailed distributions, and heteroscedastic variance assumptions. As a result of the multiple *t*-test analyses performed, a false-positive detection controlling mechanism was introduced (Genovese et al., 2002). This subsequent filter guaranteed that, on average, the false-positive detection rate was below our cutoff of 0.05.

Volume of activation was compared across experimental groups using the non-parametric Kruskall–Wallis test statistic. The brain areas were rank ordered for their significance. Brain areas were considered statistically different between experimental groups when comparison produced *P*-values less than or equal to our cutoff of 0.05.

#### Advances over previous imaging studies

There have been numerous studies using various pain-inducing stimuli to affect changes in brain activity in animals and in some cases these have been performed in awake rats as described here. For example Becerra et al. (Becerra et al., 2011) performed awake imaging in rats in response to noxious thermal stimuli and reported activation patterns across numerous brain areas. However, the present study focuses on capsaicin-induced pain mediated through the TRPV1 receptor and identifying the distributed integrated neural circuits engaged through this mechanism by using the TRPV1 knockout. The earliest fMRI study on capsaicin-induced pain in animals used a surface coil and a gradient-echo pulse sequence to image dorsal forebrain structures in alpha–chloralose anesthetized rats (Malisza and Docherty, 2001). Performed on a 9.4 T magnet, this study showed clear event related activation of the anterior cingulate and a less clearly defined anatomical area referenced as the frontal cortex. The increased BOLD signal intensity was sustained for about 2–4 min after subcutaneous injection of capsaicin before returning to baseline. The BOLD signal change could be blocked by pretreatment with morphine. Rats anesthetized with isoflurane and imaged at 7.0 T with a gradient-echo pulse sequence and surface coil localized to the somatosensory cortex showed a similar early increase in BOLD signal that could be blocked with morphine (Asanuma et al., 2008). BOLD imaging using a surface coil at 2.35 T and a spin-echo pulse sequence in isofluorane anesthetized rats reported activation in the periaqueductal gray, parabrachial nucleus, pontine reticular nucleus, hippocampus, and thalamus (Moylan Governo et al., 2006). While this study included much of the brain it was limited in spatial resolution to gross brain areas due to the relative insensitivity of fast spin echo imaging at low field strength. In a recently published paper Tsurugizawa and colleagues reported changes in brain activity in awake TRPV1 knockout mice in response to intragastric capsaicin (Tsurugizawa et al., 2013). Low field strength and a gradient echo pulse sequence limited the spatial resolution of the signal to gross areas of the brain, many of which were asymmetric, making any interpretation of well-defined integrated neural circuits difficult.

The present study, while corroborating these capsaicin-induced changes in BOLD activation, greatly extended the identification of the distributed neural circuits by circumventing many of the problems and limitations in these earlier imaging studies. First and foremost we imaged awake rats eliminating the many confounds associated with the use of anesthesia to study pain-induced changes in brain activity (Borsook and Becerra, 2011). To image the entire brain and improve field homogeneity we used a quadrature transmit/receive, volume coil. To optimize space filling and reduce the drop-off of signal due to the distance between signal source and the receive element, the coil was built into the head-holder. With an appropriately large rat head, the fit approximates the distance of a surface coil giving exceptional signal-to-noise ratio (SNR) and homogeneity over the entire brain. Lastly we performed BOLD imaging with a spin-echo pulse sequence instead of the more conventional gradient-echo sequence in optimize the neuroanatomy and resolution of the functional scans.

## Results

**Figure 3:**
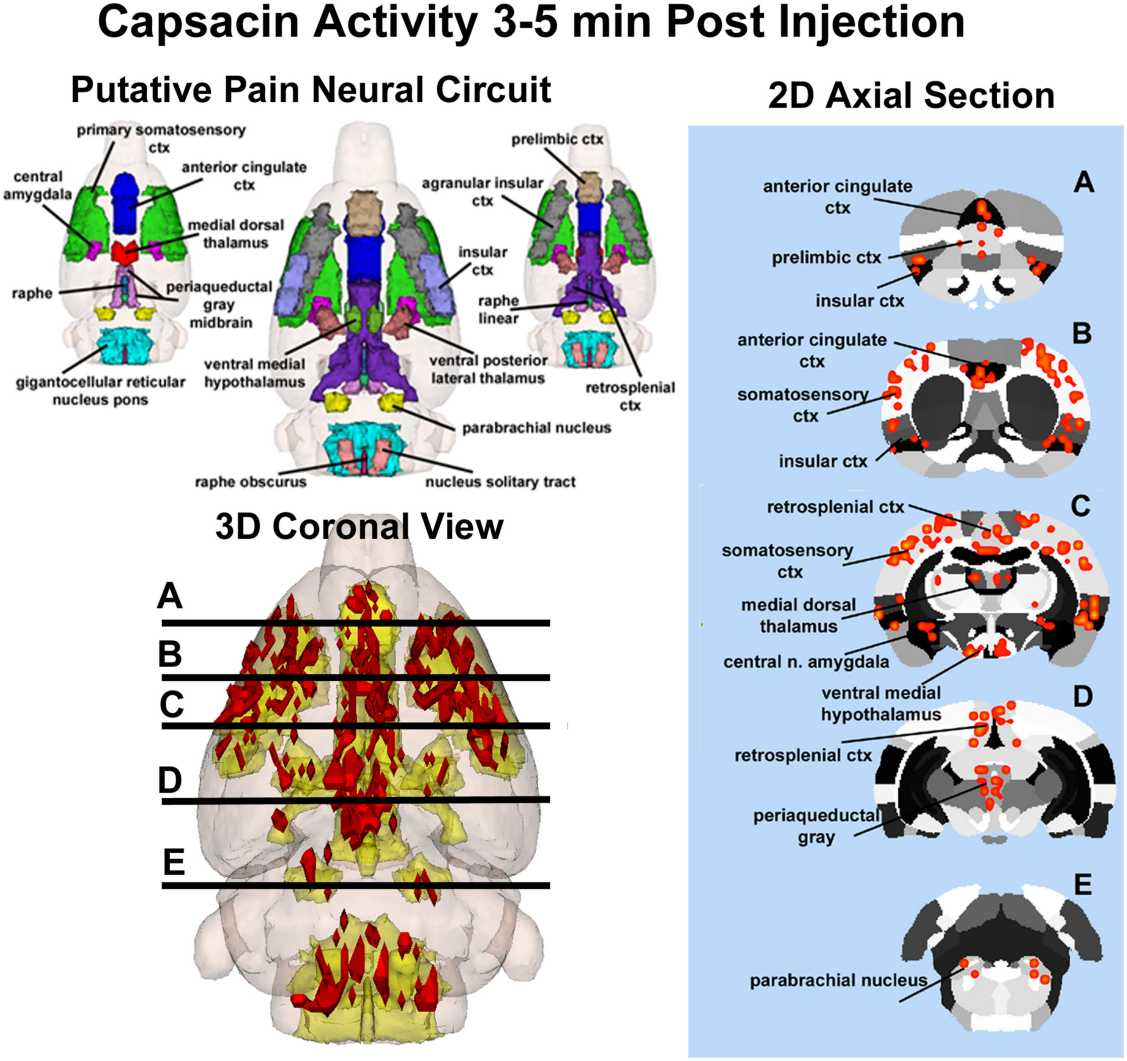
**Capsaicin-induced pain in wild-type rats**. Shown on the left is a 3D color representation of the different brain areas comprising the putative pain neural circuit of the rat. The segmented, annotated illustration is a coronal view. The yellow/gold illustration below is confluence of the segmented brain areas showing the location of the average, significant increase (red) in BOLD signal for nine rats, 3–5 min after capsaicin injection into the right hind-foot. The panel of 2D axial images on the far left depict the location of significant increase in BOLD signal (red) in brain slices approximating the positions **(A–E)** shown in the 3D illustration.

**Figure 4:**
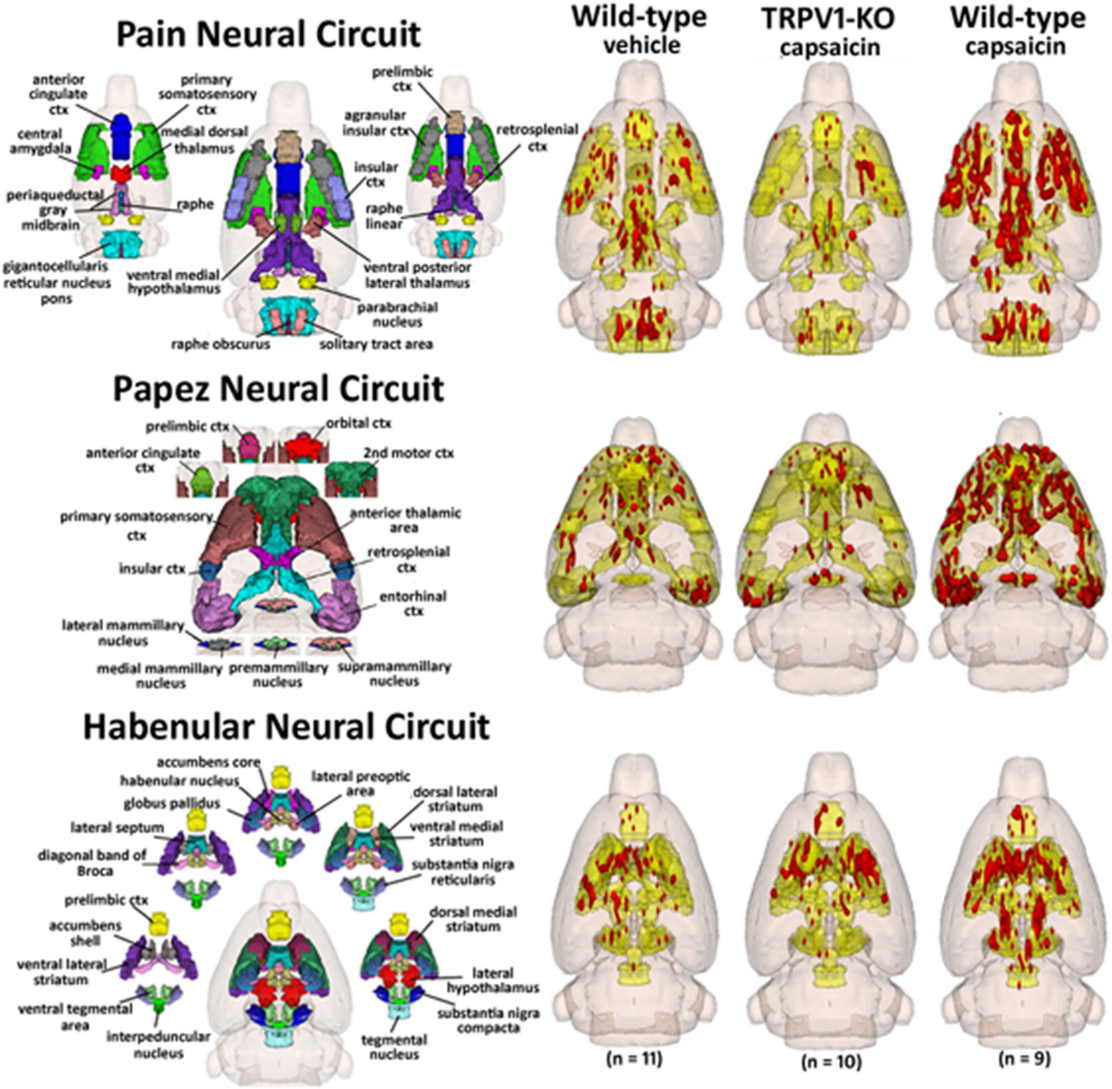
**Capsaicin pain in wild-type and TRPV1 rats**. Shown are 3D color representations of the different brain areas comprising the putative pain neural circuit (**top**), Papez cortical loop (**middle**), and the habenular system (**bottom**) of the rat. The distinct areas comprising each neural circuit have been coalesced into a single 3D volume (shown in yellow), and are presented as a 3 × 3 grid in which rows correspond to each distinct neural circuit and columns correspond to experimental conditions. In the single 3D volume of each neural circuit, the red depicts the location of the average significant increase in BOLD signal 3–5 min after vehicle injection into wild-type Sprague-Dawley rats (left column), capsaicin injection into TRPV1-KO rats (middle column), and capsaicin injection into wild-type Sprague–Dawley rats (right column).

Capsaicin injection into the hindpaw of Sprague–Dawley wild-type control rats activated many of the areas in the putative neural circuit of pain, as depicted in Figure 3. Activation maps depicting the location of the positive BOLD signal change in a 3D rendering of the pain neural circuit (yellow), or in 2D axial sections from the rat atlas, illustrate the regions activated by capsaicin in wild-type rats. The pain neural circuit was adopted from Gauriau and Bernard using the parabrachial nucleus as key node (Gauriau and Bernard, 2002). Table 1 lists all of the brain areas that are significantly activated in wild-type controls following capsaicin injection as compared to vehicle. This truncated list is taken from 137 brain areas rank ordered by statistical significance (see Supplementary Materials for full Table 1S). Of the 17 brain areas comprising the putative neural circuit for pain, 11 are significantly activated by capsaicin injection as shown highlighted in yellow. The gigantocellular reticular nucleus of the pons trends toward being significant (*p* < 0.064). The central nucleus of the amygdala, a key brain area involved in pyschogenic stress and fear appears atop the list. The limbic cortex consisting of the anterior cingulate, retrosplenial, infralimbic, and insular cortices are also represented as is the subiculum, dentate gyrus, and CA3 of the hippocampus and the paraventricular, mediodorsal and laterodorsal nuclei of the thalamus. The limbic cortex and its connections to the dorsal thalamus, hippocampus and amygdala comprise a distributed integrated neural circuit involved in the processing and memory of emotional experience. Shown in Figure 4 is a 3D rendering of the cortical loop of Papez and the activation therein following vehicle injection in wild-type Sprague–Dawley control rats, capsaicin in TRPV1-KO rats, and capsaicin in wild-type controls.

**Table 1:**
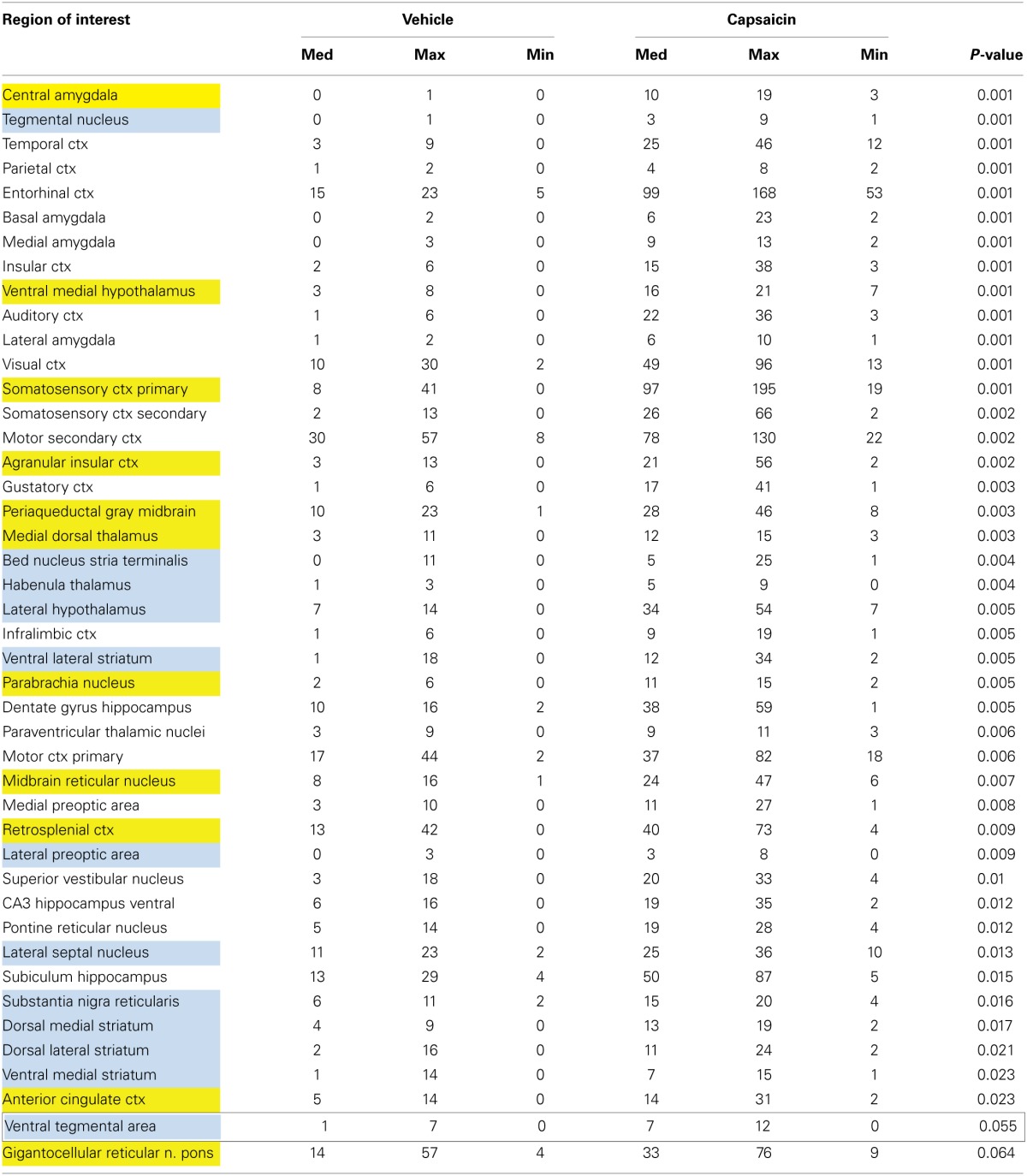
**Volume of activation—positive BOLD in wild-type**.

*Shown is a truncated list of 137 brain areas and their median (med), maximum (max) and minimum (min) number of voxels activated at 3–5 min post-subdermal injection of vehicle and capsaicin. The brain areas were rank ordered for their statistical significance; the box delineates significant areas. Brain areas were compared across experimental groups using a non-parametric Kruskall–Wallis test statistic. Brain areas were considered different between experimental groups if P-values were less than or equal to our cutoff of 0.05. P-values are presented on the far right column. The yellow highlight denotes brain areas comprising the putative neural network of pain. Areas highlighted in blue comprise the putative neural network of the habenular system*.

Table 2 lists all of the brain areas that are significantly activated in TRPV1-KO rats following formalin injection. Formalin, which activates TRPA1 pain receptors, was used as a positive control. This truncated list is taken from 137 brain areas, rank ordered by statistical significance (see Supplementary Materials for full Table 2S). There was only modest activation in the vehicle and capsaicin groups as indicated by the median number of voxels highlighted in gray. Formalin significantly activated the somatosensory cortex, periaqueductal gray, and medial dorsal thalamus of the pain neural circuitry shown in yellow. The prelimbic cortex showed a trend toward significance (*P* < 0.057). Activation in the cerebellum was also significantly different across experimental groups. Note the activation of the habenular nucleus. The habenular nucleus of the thalamus and its connections with basal ganglia, e.g., striatum, ventral tegmental area, ventral pallidum, and accumbens are part of the habenular neural circuit involved with anticipation and prediction of aversive events. Many areas associated with the habenular system were significantly activated or trended toward significance (blue highlight). The activation of the habenular system was also observed in capsaicin-injected wild-type controls as seen in Table 1 highlighted in blue. A 3D rendering of the habenular system and the activation therein following vehicle and capsaicin injection is provided in Figure 4.

**Table 2:**
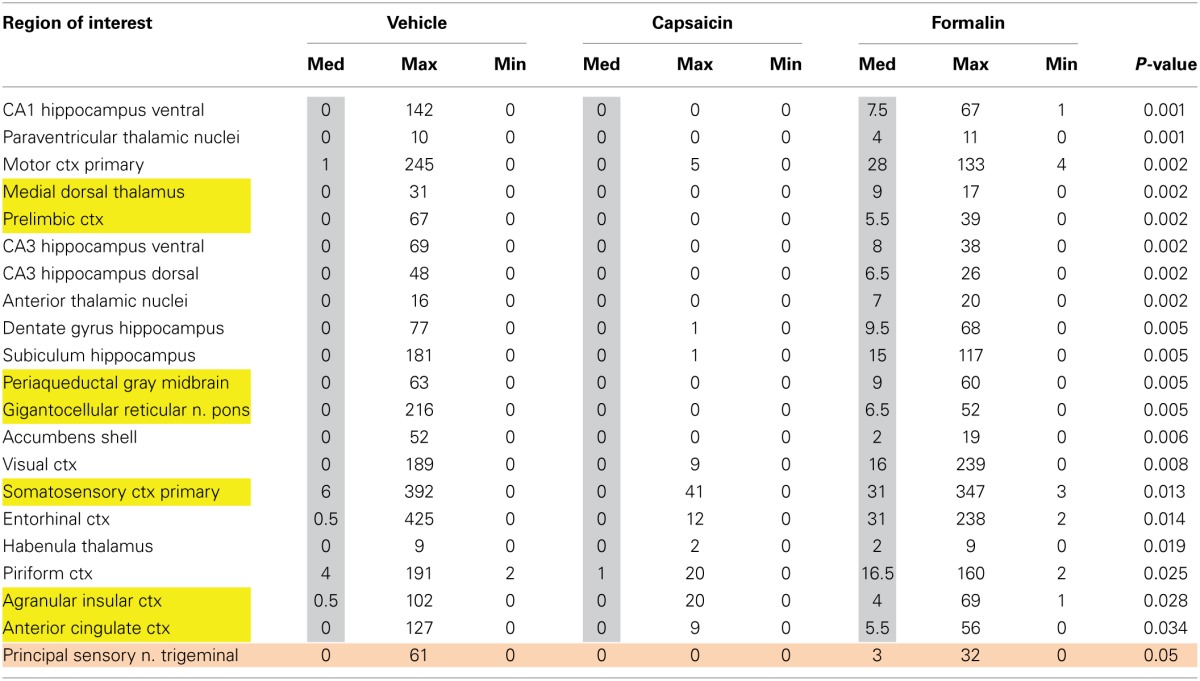
**Volume of activation—positive BOLD in TRPV1 knockout**.

*Shown is a truncated list of 137 brain areas and their median (med, gray highlight), maximum (max) and minimum (min) number of voxels activated at 3–5 min post-intradermal injection of vehicle, capsaicin and formalin into the hindpaw of TRPV1 knockout rats. The brain areas were rank ordered for their significance; the tan highlight delineates significant areas. Brain areas were compared across experimental groups using a nonparametric Kruskall–Wallis test statistic. Brain areas were considered different between experimental groups if P-values were less than or equal to our cutoff of 0.05. P-values are presented on the far right column. The yellow highlight denotes brain areas comprising the putative neural network of pain*.

Second-order post-hoc analysis was performed to examine pairwise differences between the responses to vehicle and capsaicin in TRPV1-KO rats. Table 3 lists all of the brain areas that exhibit significant differences in activation. In TRPV1-KO rats, vehicle treatment resulted in greater activation in 18 brain regions in comparison to capsaicin treatment. Of these, 4 regions are part of the putative pain neural circuit. A fifth region that is also part of the putative pain neural circuit, the gigantocellular reticular nucleus of the pons, displayed a trend toward significance (*P* < 0.053).

**Table 3:**
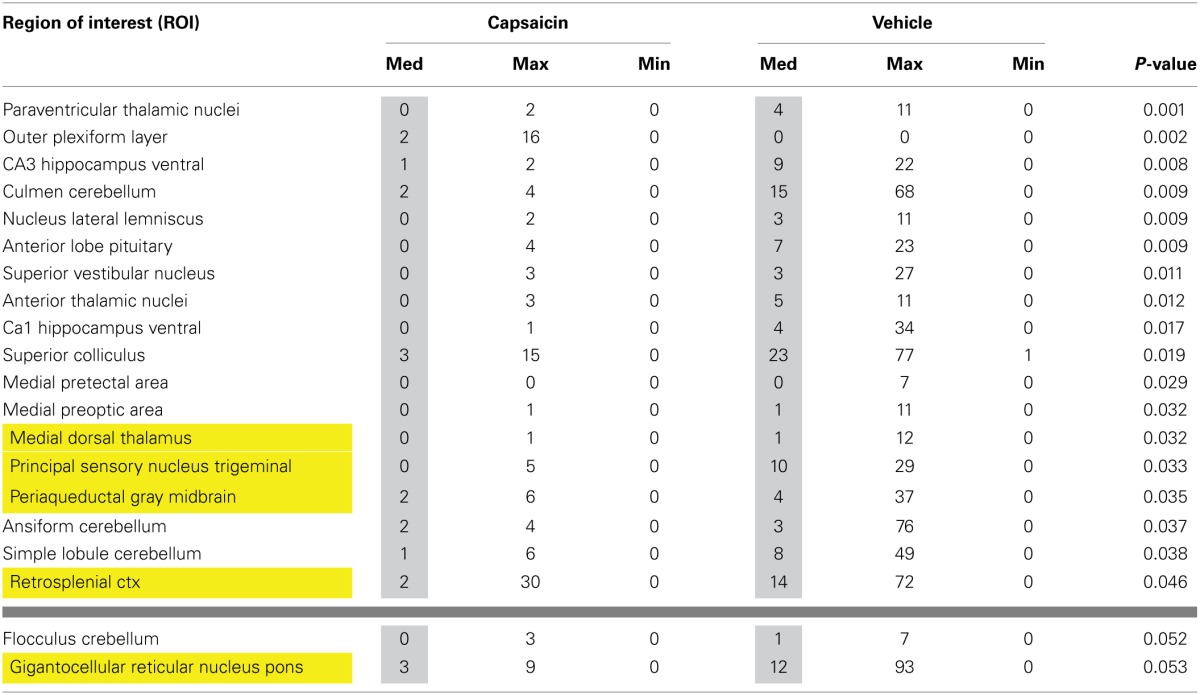
**Volume of activation for positive BOLD in TRPV1 knockout following vehicle and capsaicin**.

*Shown is a truncated list of 137 brain areas and their median (med, gray highlight), maximum (max) and minimum (min) number of voxels activated at 3–5 min post-intradermal injection of vehicle and capsaicin into the hindpaw of TRPV1 knockout rats. The brain areas were rank ordered for their significance; the gray line delineates significant areas. The yellow highlight denotes brain areas comprising the putative neural network of pain*.

**Figure 5:**
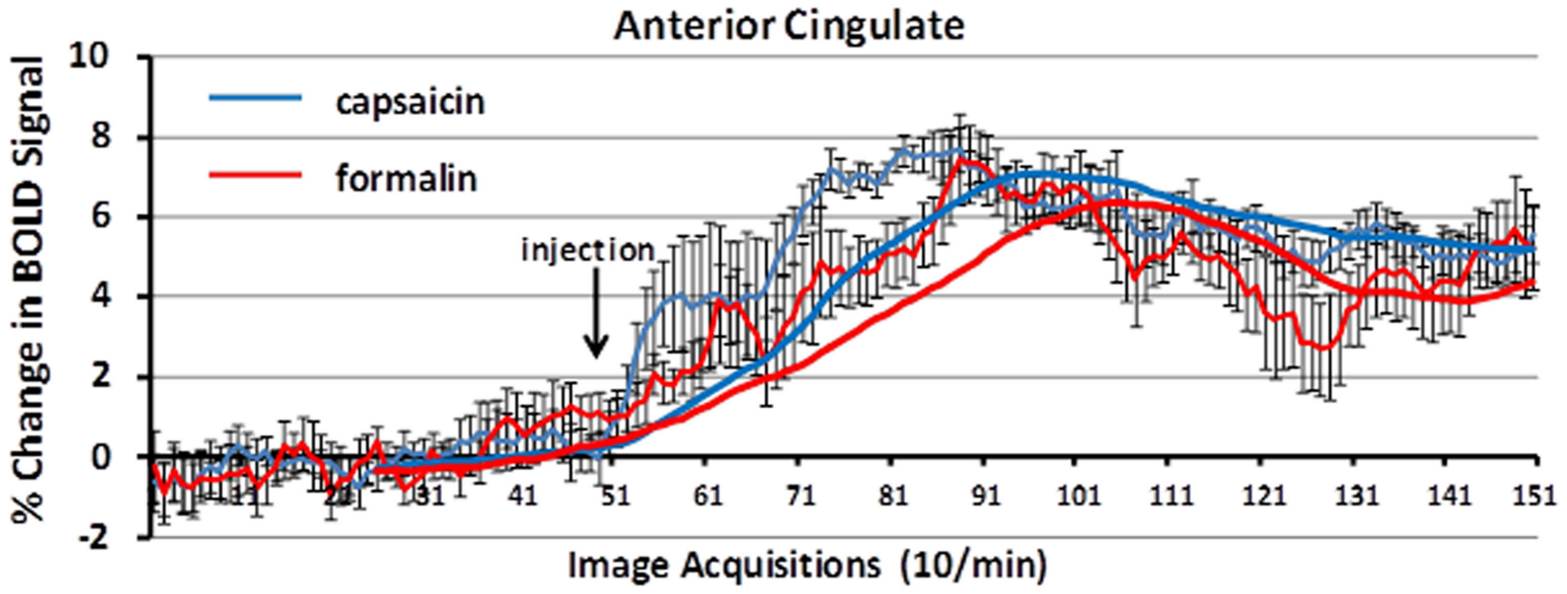
**BOLD signal change over time**. Shown is the percent change in BOLD signal over a 10 min period (acquisitions 51–150) following capsaicin in wild-type controls (blue) and formalin injection in TRPV1 KO rats (red). The anterior cingulate cortex, a key area in the pain neural circuit, shows a maximal change in BOLD signal within 3–5 min post-injection of capsaicin or formalin. Trend lines (solid blue and red) represent simple moving averages over 25 timepoints. Vertical lines denote SEM.

Figure 5 shows the change in BOLD signal over time in the anterior cingulate cortex following capsaicin injection in wild-type controls and formalin injection in TRPV1-KO rats. The time-courses for both stimuli are very similar, with a peak in the percent change in BOLD signal occurring 3–4 min post-injection.

**Figure 6:**
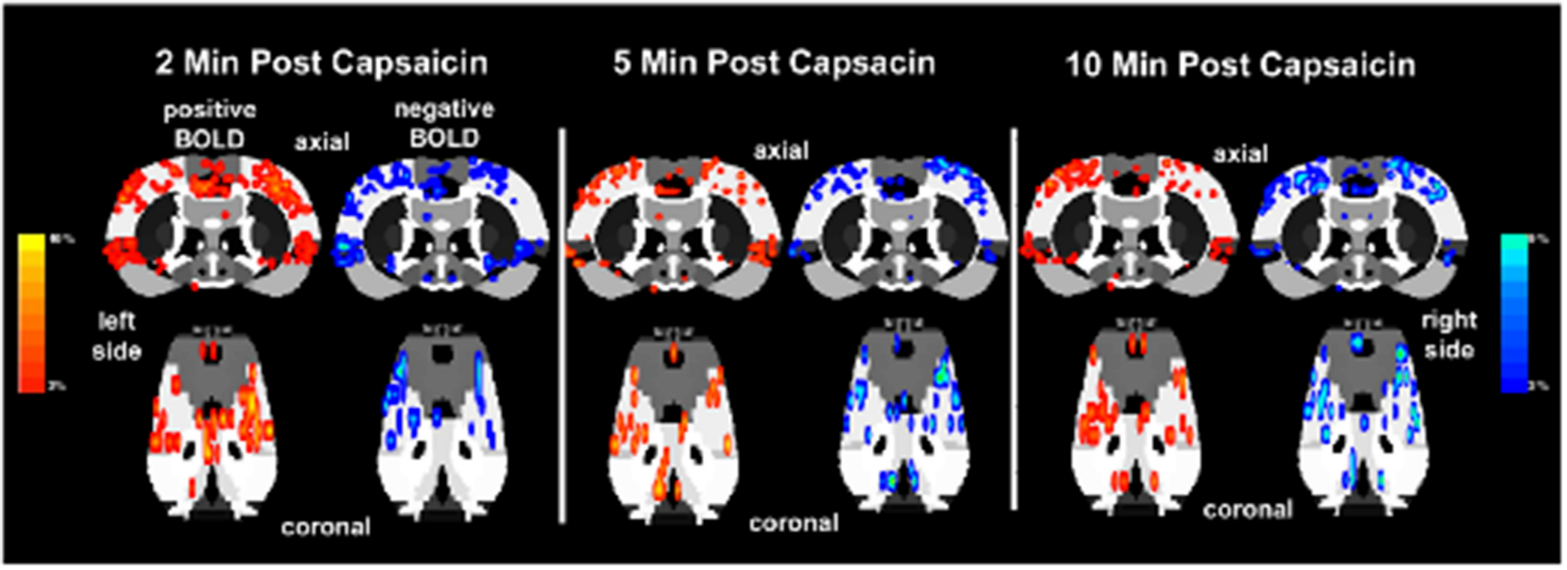
**Lateralization of BOLD signal change over time**. Shown below are activation maps of positive and negative BOLD signal following capsaicin injection into the right hindpaw of wild-type Sprague–Dawley rats (*n* = 9). The contralateral (left side) somatosensory cortex is circled in yellow for each time point. There is no clear lateralization of positive BOLD in the first 2 min of data acquisition. Instead there appears to be more negative BOLD as compared to the ipsilateral somatosensory cortex (right side). However, by 5 and 10 min post-capsaicin the contralateral side presents with more positive BOLD signal. Areas of activation have been smoothed. Colored BOLD activation scales indicate percent changes in positive BOLD (red spectrum) and negative BOLD (blue spectrum).

The lateralization of the BOLD signal change following capsaicin injection into the right hindpaw was not fixed over the 10 min imaging period post-stimulus as shown in Figure 6. It was expected that activation would be greatest in the left contralateral hemisphere of the brain. At 2 min post-injection both positive and negative BOLD changes appear to be bilateral. At the 5 and 10 min mark, however, the lateralization becomes clearer. This is also accompanied by an increase in negative BOLD signal in the right hemisphere.

## Discussion

Functional neuroimaging of BOLD responses to painful stimuli in un-anesthetized, awake animals identified brain regions that have previously been shown to mediate neural responses to pain, corroborating past work with a novel experimental approach. Furthermore, our data suggest that neuroimaging of pain in awake, conscious animals has the potential to inform the neurobiological basis of full and integrated perceptions of pain which, as we show here, recruit both affective and learning systems in addition to areas that subserve pain sensation.

Capsaicin injection in awake wild-type rats activated the parabrachial nucleus and both major nociceptive pathways, which include somatosensory areas involved in sensory discrimination of painful stimuli, and a pathway linking the brainstem with hypothalamus, midline thalamic areas and limbic cortex involved in attentional and motivational aspects of pain (see Table 1). Along with the gigantocellular reticular area, which displayed a statistical trend of activation in response to capsaicin in wild-type rats, the parabrachial nucleus has monosynaptic connections to the periaqueductal gray, ventral medial hypothalamus, central nucleus of the amygdala, ventral medial and midline thalamic nuclei (Hylden et al., 1989; Bernard et al., 1993; Bester et al., 1995, 1997; Jasmin et al., 1997; Bourgeais et al., 2001a). The projection of these areas to the forebrain cortex may function in the motivational and attentional aspects of pain.

In addition to areas typically thought to comprise the putative pain neural circuit, we observed capsaicin-induced activation in limbic and habenular regions. The central nucleus of the amygdala, a key limbic region involved in pyschogenic stress and fear appears atop the list of capsaicin-activated regions. Areas of the limbic cortex which included anterior cingulate, retrosplenial, insular, infralimbic, and orbital cortices, and which comprise the Papez circuit of emotional experience, were also activated in response to capsaicin in wild-type control rats. The Papez circuit connects the hypothalamus and hippocampus to the limbic cortex through the midline dorsal thalamic nuclei e.g., anterior, mediodorsal, paraventricular nuclei (Papez, 1937). These areas send primary projections to the anterior cingulate, retrosplenial, prefrontal, orbital, and somatosensory cortices. Involvement of the Papez circuit suggests that conscious processing of TRPV1-mediated pain responses recruits brain regions that integrate sensory and attentional information with affective information. The habenular system was also activated in response to capsaicin administration in wild-type controls. The habenular system is associated with anticipation and prediction of aversive events (Matsumoto and Hikosaka, 2007; Hikosaka et al., 2008). The influence of the habenular system on aversive learning is mediated, in part, by controlling the activity of dopaminergic neurons in the midbrain and the subsequent release of dopamine in the striatum and prefrontal cortex (Matsumoto and Hikosaka, 2007; Lecourtier et al., 2008). Thus, in addition to corroborating results from past studies, the neuroimaging of pain in awake rats elucidated brain regions that may contribute to the affective and aversive learning aspects of pain sensing.

Second-order *post-hoc* analyses revealed unexpected differences in activation between vehicle and capsaicin treatment in TRPV1-KO rats. We expected the response to vehicle and capsaicin administration to be equivalent in rats lacking the TRPV1 receptor since capsaicin-induced nociception is mediated through binding at the TRPV1 receptor. However, as shown in Table 3, in rats lacking TRPV1, vehicle administration (in comparison to capsaicin administration) resulted in greater positive BOLD response in several brain regions, including those in the putative pain neural circuit, indicating that capsaicin may have antinociceptive properties independent of TRPV1 receptor binding. Capsaicin has been previously demonstrated to have antinociceptive properties under certain conditions (Szallasi and Blumberg, 1999; Brederson et al., 2013). However, existing theories of capsaicin-induced antinociception predominantly rely on the notion that continued binding of capsaicin at the TRPV1 receptor eventually results in a refractory state whereby the receptor is desensitized to further capsaicin exposure (Szallasi and Blumberg, 1999). Capsaicin has been proposed to exhibit properties independent of TRPV1 binding through direct inhibition of mitochondrial respiration (Athanasiou et al., 2007; Bley et al., 2012) and this mechanism may also be responsible for the antinociceptive effects observed in this study. However, future work should consider the possibility that capsaicin binds to other receptors that mediate pain relief especially since it has been shown that anandamide, an endocannabinoid that binds to the cannabinoid 1 (CB1) receptor to produce analgesic effects, can also bind to the TRPV1 receptor (Zygmunt et al., 1999; van der Stelt et al., 2005; Vriens et al., 2009). Future work on TRPV1-independent antinociceptive effects of capsaicin may be pertinent to drug development for pain relief.

While capsaicin administration in wild-type rats was capable of eliciting robust activation in the putative pain neural circuit, Papez circuit, and the habenular system, rats lacking the TRPV1 receptor displayed minimal activation in these brain regions in response to capsaicin. As Figure 4 illustrates, capsaicin administration to TRPV1-KO rats produced BOLD activation equivalent to or less than saline vehicle administration to wild-type control rats. These data demonstrate that expression of the TRPV1 receptor is necessary for the perception of capsaicin-induced pain, and provide functional verification of the absence of TRPV1 expression in the transgenic rat model used in this study. Furthermore, failure to express the TRPV1 receptor throughout the lifespan did not result in the recruitment of compensatory mechanisms to facilitate lost capsaicin binding since adult TRPV1-KO rats did not experience BOLD activation in response to capsaicin injection. Pain induction through administration of formalin, mediated through TRPA1 receptor binding, resulted in BOLD activation in brain regions included in the putative pain neural circuit, demonstrating the ability of this transgenic model to cause specific disruption of TRPV1-mediated pain. Examination of the time course of BOLD activation provides a depiction of how the processing of painful stimuli unfolds in the brain. Figure 5 illustrates the change in BOLD signal in the anterior cingulate cortex over the course of the 10 min imaging session following administration of capsaicin in wild-type rats and formalin in TRPV1-KO rats. Maximal change in BOLD signal peaks 3–5 min following the injection of both capsaicin in wild-type rats and formalin in TRPV1-KO rats, illustrating the similarity in the temporal dynamics of the BOLD activation. Taken together these data validate the usage of the TRPV1-KO rat for the experimental study of pain.

Lateralization of BOLD signal across the imaging session was not fixed, and was not yet present 2 min following injection of capsaicin. Indeed, dynamic fluctuations in negative and positive BOLD signal occur across the cerebral hemispheres within the first 1–2 min of stimulation (as shown in Figure 6). These fluctuations in BOLD signal appeared to stabilize between 3 and 5 min. Interestingly, rapid fluctuation of BOLD signal across hemispheres in response to a noxious forepaw stimulus in anesthetized rats has been reported previously (Tuor et al., 2000). However, 5 min following capsaicin administration, wild-type rats displayed lateralization of BOLD signal changes with greater activation in the contralateral hemisphere, as expected. Imaging neural responses to painful stimuli in awake, conscious animals revealed a lateralized pattern of activational changes that unfolded over the timecourse of the imaging session.

### Caveats and data interpretation

For any imaging study on awake animals the issues and consequences related to the stress of head restraint and restricted body movement must be considered. Protocols have been developed to help lessen the stress of an imaging study by acclimating animals to the environment of the MR scanner and the restraining devices. Such procedures have been shown in past studies to reduce stress hormone levels and measures of sympathetic autonomic activity (Zhang et al., 2000; King et al., 2005). Albeit reduced due to acclimation procedures, it is possible that the stress associated with head restraint and restricted body movement could result in a ceiling effect with respect to BOLD activation in areas that mediate the perception of pain. We did not find this to be the case—robust differences in BOLD activation in many regions were observed between saline-treated and capsaicin-treated wild-type rats, as illustrated in Table 1. This provides strong evidence that the measures required to perform awake imaging do not rule out the study of pain.

For this experiment, we used a newly developed transgenic rat (SAGE Labs, St. Louis) containing a biallelic deletion of the TRPV1 gene. Details on the validation of the model are provided online^1^. Judging from the Western blot in which the knockout shows a faint band near the TRPV1 size, it is possible that the knockout model produced hypomorphic mutants that express TRPV1, though at an exceedingly low level, or dominant-negative mutants that express a truncated and functionally antagonistic form of the protein. Here, we demonstrate that this model, regardless of whether it lacks TRPV1 completely or predominantly, fails to respond to capsaicin, the natural ligand for TRPV1. Future work that precisely characterizes the genetics of this model will shed light on the degree to which results obtained here are due to hypomorphic or dominant-negative mutations.

The injection of formalin to TRPV1 KO rats in this study showed a high degree of overlap with previous work elsewhere on formalin induction of BOLD responses in wild-type rats (Shih et al., 2008), activating regions such as the cingulate cortex, hippocampus, somatosensory cortex, motor cortex, visual cortex, and medial dorsal thalamus. However, the use of formalin as a positive control in TRPV1 KO rats was not as robust as we initially anticipated since only four of the 17 areas in the putative neural circuit of pain were activated. Differences in several experimental parameters may have contributed to the differences found here. First, the current study used a 3% concentration of formalin instead of 5% as described in some other studies (Shih et al., 2008). Second, it is possible that formalin acting through the TRPA1 receptor activates brain areas processing pain that have similarities to capsaicin but are different enough to require their own pain “finger print.” Indeed, the areas of activation observed in Table 2 are not unlike those reported by Morrow and coworkers using quantitative autoradiography to assess regional cerebral blood flow in awake Sprague–Dawley rats in response hindpaw injection of 2.5% formalin (Morrow et al., 1998). The somatosensory cortex, midbrain periaqueductal gray, midline thalamic nuclei, parietal cortex, and hippocampus all showed activation over controls. Interestingly, they reported activation of the habenula and interpenduncular nucleus, key nodes in the habenular system that are also reported here. Third, if future work reveals that this transgenic model produced a functionally antagonistic dominant-negative mutant, this may explain the weak effect of formalin since TRPV1 and TRPA1 receptors are functionally coupled in dorsal root ganglia neurons (Forster et al., 2009). Lastly, the response to formalin is known to be biphasic, with periods of peak activity separated by a period of quiescence. Individual differences in the timing of the quiescent period may have resulted in lower levels of BOLD activation overall since analysis was performed on data collected from all animals 3–5 min following injection when some animals may have already started to experience quiescence.

Animals underwent different injections and imaging sessions separated by 2 weeks. Incorporating this delay into the experimental approach was meant to minimize the confound of repeated exposure to noxious pain. However, future studies may reveal that acute pain stimulation results in long-lasting changes in, for example, receptor expression that may have contributed to the results. Future imaging studies on formalin-induced pain will further reveal the temporal-spatial pattern of neural response to stimulation of TRPA1 receptors by formalin.

### Conflict of interest statement

Ferris, Kulkarni and Nedelman have a financial interest in Ekam Imaging the company that supported the research. Gamble and Simmons have a financial interest in SAGE Labs the company that supported the research. The authors declare that the research was conducted in the absence of any commercial or financial relationships that could be construed as a potential conflict of interest.

## Footnotes

^1^Pain Knockout Rat Models: *In Vivo* Model for Pain Research [Internet]. St. Louis, SAGE Labs, Inc. ©2013 [cited 2015 Jan 6]. Available online at: http://www.sageresearchlabs.com/files/Pain%20Flyer_digital.pdf
