# Supplementary material for: Identifying the integrated neural networks involved in capsaicin-induced pain using fMRI in awake TRPV1 knockout and wild-type rats
Source: Front Syst Neurosci. 2015 Feb 19;9:15. doi: 10.3389/fnsys.2015.00015 (PMC4333803; doi:10.3389/fnsys.2015.00015)
Supplement: Supplementary file 1 [file DataSheet1.DOCX]

***Supplementary Material***

**Identifying the Integrated Neural Networks Involved in Capsaicin-Induced Pain Using fMRI in Awake TRPV1 Knockout and Wild-Type Rats**

**Jason R. Yee^1^, William Kenkel^1^, John C. Caccaviello^1^, Kevin Gamber^2^, Phil Simmons^2^, Mark Nedelman^3^, Praveen Kulkarni^1^, Craig F. Ferris^1^**

^1^Northeastern Univ., Center for Translational NeuroImaging, Boston, MA;

^2^SAGE Labs, St Louis, MO;

^3^Ekam Imaging, Boston, MA

Corresponding author:

Craig F. Ferris, Department of Psychology, Center for Translational NeuroImaging, Northeastern University, Boston, Massachusetts 02115-5000, Email: [c.ferris@neu.edu](mailto:c.ferris@neu.edu),

Phone: (617) 373-3083

**1. Supplementary methods describing composite activation maps**

A statistical composite of all subjects from each treatment group was created. The composite statistics were built using the inverse transformation matrices. Each composite pixel location (ie, row, column, and slice) was pre-multiplied by [*T_i_*]^-1^, and mapped within a voxel of subject (*i*). A tri-linear interpolation of the subject’s voxel values (percentage change) determined the statistical contribution of the subject (*i*) to the composite (row, column, and slice) location. The use of [*T_i_*]^-1^ ensured that the full volume set of the composite was populated with subject contributions. The median value from all subjects within the group were used to determine the composite value. The median number of activated pixels that had highest contribution from group in a particular ROI were displayed in a composite map. Activated composite pixels were calculated as follows:

$$Number of Activated composite voxels at {ROI}_{j}=median{(Activated Pixels {subject}_{i} at {ROI}_{j})}_{i=1}^{N}$$

The composite percent change for the time-history graphs for each region was based on the weighted average of each subject, as follows:

where *N* was number of subjects.

**2. Supplementary Table comparing capsaicin injection in TRPV1-KO vs. Wild-type rats**

**Table 4**

|  | **TRPV1-KO** | | | **Wild-type** | | |  |
| --- | --- | --- | --- | --- | --- | --- | --- |
| **Region of Interest (ROI)** | **med** | max | min | **med** | max | min | **P value** |
| trigeminal nerve | **0** | 1 | 0 | **6** | 13 | 3 | **0** |
| parietal ctx | **0** | 1 | 0 | **4** | 8 | 2 | **0** |
| paraventricular thalamic nuclei | **0** | 2 | 0 | **9** | 11 | 3 | **0** |
| medial dorsal thalamus | **0** | 1 | 0 | **12.5** | 18 | 3 | **0** |
| superior vestibular nucleus | **0** | 3 | 0 | **20** | 33 | 4 | **0** |
| auditory ctx | **0** | 2 | 0 | **23** | 36 | 3 | **0** |
| ansiform cerebellum | **0** | 4 | 0 | **47.5** | 98 | 16 | **0** |
| anterior lobe pituitary | **0** | 4 | 0 | **23** | 46 | 9 | **0** |
| somaotsensory ctx secondary | **0** | 1 | 0 | **24** | 66 | 2 | **0** |
| posterior hypothalamus | **0** | 2 | 0 | **8** | 16 | 1 | **0** |
| nucleus lateral lemniscus | **0** | 2 | 0 | **9.5** | 21 | 3 | **0** |
| granular cell layer | **0** | 8 | 0 | **37** | 80 | 11 | **0** |
| lateral septal nucleus | **0** | 5 | 0 | **27.5** | 36 | 10 | **0** |
| pontine reticular nucleus | **0** | 2 | 0 | **18.5** | 28 | 4 | **0** |
| cortical amygdala | **0** | 5 | 0 | **22.5** | 33 | 11 | **0** |
| principal sensory nucleus trigeminal | **0** | 5 | 0 | **19.5** | 41 | 8 | **0** |
| paraflocculus cerebellum | **0** | 9 | 0 | **34** | 51 | 16 | **0** |
| medial preoptic area | **0** | 1 | 0 | **11.5** | 27 | 1 | **0** |
| periaqueductal gray midbrain | **2** | 6 | 0 | **29** | 46 | 8 | **0** |
| simple lobule cerebellum | **1** | 6 | 0 | **63.5** | 93 | 14 | **0** |
| temporal ctx | **1** | 6 | 0 | **25.5** | 46 | 12 | **0** |
| cerebellar peduncle | **2** | 9 | 0 | **59** | 204 | 17 | **0** |
| culmen cerebellum | **2** | 4 | 0 | **76.5** | 110 | 25 | **0** |
| inferior colliculus | **14** | 21 | 0 | **56** | 74 | 22 | **0** |
| insular ctx | **0** | 3 | 0 | **14** | 38 | 3 | **0** |
| motor ctx primary | **8** | 15 | 0 | **35.5** | 82 | 18 | **0** |
| entorhinal ctx | **11** | 42 | 6 | **100** | 168 | 53 | **0** |
| parabrachial nucleus | **0** | 5 | 0 | **10.5** | 15 | 2 | **0** |
| gigantocellular reticular nucleus pons | **3** | 9 | 0 | **40** | 76 | 9 | **0** |
| central lobule cerebellum | **2** | 22 | 0 | **75.5** | 114 | 22 | **0** |
| medial amygdala | **1** | 2 | 0 | **9** | 13 | 2 | **0** |
| anterior thalamic nuclei | **0** | 3 | 0 | **8.5** | 14 | 3 | **0** |
| CA1 hippocampus ventral | **0** | 1 | 0 | **20.5** | 39 | 1 | **0** |
| somatosensory ctx primary | **6** | 32 | 1 | **94** | 195 | 19 | **0** |
| CA3 hippocampus ventral | **1** | 2 | 0 | **20** | 35 | 2 | **0** |
| dorsal striatum dorsal lateral | **0** | 2 | 0 | **11** | 24 | 2 | **0** |
| bed nucleus stria terminalis | **0** | 1 | 0 | **7.5** | 25 | 1 | **0** |
| CA3 hippocampus dorsal | **1** | 2 | 0 | **12.5** | 23 | 2 | **0** |
| interpeduncular nucleus | **0** | 2 | 0 | **2.5** | 8 | 1 | **0** |
| visual ctx | **2** | 23 | 0 | **46** | 96 | 13 | **0** |
| central amygdala | **2** | 4 | 0 | **9** | 19 | 3 | **0** |
| neural lobe pituitary | **0** | 2 | 0 | **5.5** | 10 | 2 | **0** |
| anterior hypothalamus | **0** | 1 | 0 | **3.5** | 10 | 1 | **0** |
| outer plexiform layer | **2** | 16 | 0 | **40** | 84 | 6 | **0** |
| piriform ctx | **6** | 32 | 2 | **80.5** | 127 | 27 | **0** |
| dorsal striatum ventral medial | **0** | 2 | 0 | **9** | 15 | 1 | **0** |
| glomerular layer | **0** | 26 | 0 | **46** | 83 | 6 | **0.001** |
| motor secondary ctx | **6** | 26 | 0 | **77.5** | 130 | 22 | **0.001** |
| dorsal striatum ventral lateral | **2** | 4 | 0 | **16** | 34 | 2 | **0.001** |
| medial septum | **0** | 5 | 0 | **6** | 11 | 1 | **0.001** |
| tenia tecta ctx | **1** | 10 | 0 | **17.5** | 35 | 3 | **0.001** |
| orbital ctx | **1** | 2 | 0 | **5** | 29 | 1 | **0.001** |
| midbrain reticular nucleus | **2** | 16 | 0 | **26.5** | 47 | 6 | **0.001** |
| flocculus crebellum | **0** | 3 | 0 | **11.5** | 19 | 0 | **0.001** |
| gustatory ctx | **1** | 3 | 0 | **17** | 41 | 1 | **0.001** |
| anterior pretectal nucleus | **0** | 0 | 0 | **1** | 8 | 0 | **0.001** |
| lateral posterior thalamus | **0** | 0 | 0 | **3.5** | 10 | 0 | **0.001** |
| lateral dorsal thalamus | **0** | 2 | 0 | **3.5** | 5 | 0 | **0.001** |
| anterior olfactory nucleus | **0** | 2 | 0 | **15** | 25 | 0 | **0.001** |
| midbrain reticular nucleus retrorubral area | **0** | 6 | 0 | **10** | 18 | 1 | **0.001** |
| central nucleus thalamus | **0** | 1 | 0 | **2** | 5 | 0 | **0.001** |
| dentate gyrus hippocampus | **2** | 25 | 0 | **36** | 59 | 1 | **0.001** |
| anterior cingulate ctx | **3** | 5 | 0 | **17** | 31 | 2 | **0.001** |
| habenula thalamus | **0** | 3 | 0 | **5** | 9 | 0 | **0.001** |
| lateral hypothalamus | **2** | 23 | 0 | **35** | 54 | 7 | **0.001** |
| basal amygdala | **1** | 3 | 0 | **7** | 23 | 2 | **0.001** |
| ventral medial hypothalamus | **1** | 17 | 0 | **16** | 21 | 7 | **0.002** |
| ventral pallidum | **0** | 1 | 0 | **7.5** | 17 | 0 | **0.002** |
| olfactory tubercles | **3** | 20 | 1 | **42** | 66 | 2 | **0.002** |
| agranular insular ctx | **3** | 7 | 0 | **20.5** | 56 | 2 | **0.002** |
| retrosplenial ctx | **2** | 30 | 0 | **35.5** | 73 | 4 | **0.002** |
| substantia nigra reticularis | **1** | 11 | 0 | **15** | 23 | 4 | **0.002** |
| dorsal striatum dorsal medial | **2** | 10 | 0 | **12** | 19 | 2 | **0.002** |
| tegmental nucleus | **0** | 2 | 0 | **3** | 9 | 1 | **0.002** |
| subiculum hippocampus | **2** | 26 | 0 | **53** | 87 | 5 | **0.002** |
| strial fundus | **0** | 1 | 0 | **3** | 5 | 0 | **0.002** |
| pontine gray | **0** | 7 | 0 | **10.5** | 19 | 2 | **0.002** |
| nucleus brachium | **0** | 3 | 0 | **2** | 4 | 1 | **0.002** |
| medial pretectal area | **0** | 0 | 0 | **2** | 2 | 0 | **0.002** |
| lateral geniculate | **0** | 1 | 0 | **2** | 5 | 0 | **0.003** |
| cochlear nucleus | **2** | 7 | 0 | **13** | 25 | 1 | **0.003** |
| posterior thalamus | **0** | 0 | 0 | **1** | 4 | 0 | **0.003** |
| red nucleus | **0** | 0 | 0 | **2** | 4 | 0 | **0.003** |
| olivary nucleus | **1** | 12 | 0 | **11.5** | 22 | 3 | **0.003** |
| zona incerta | **0** | 2 | 0 | **3** | 7 | 0 | **0.004** |
| ventral tegmental area | **0** | 5 | 0 | **7.5** | 12 | 0 | **0.004** |
| diagonal band of Broca | **0** | 1 | 0 | **6** | 12 | 0 | **0.005** |
| lateral [preoptic area | **0** | 1 | 0 | **3** | 8 | 0 | **0.006** |
| CA1 hippocampus dorsal | **1** | 11 | 0 | **14** | 32 | 0 | **0.006** |
| raphe central | **0** | 0 | 0 | **1** | 2 | 0 | **0.007** |
| raphe magnus | **0** | 0 | 0 | **1** | 3 | 0 | **0.007** |
| ventral medial thalamus | **0** | 0 | 0 | **1** | 8 | 0 | **0.008** |
| medial mammillary hypothalamus | **0** | 7 | 0 | **5.5** | 15 | 0 | **0.01** |
| trapezoid body | **0** | 4 | 0 | **3** | 10 | 0 | **0.012** |
| infralimbic ctx | **0** | 9 | 0 | **9** | 19 | 1 | **0.014** |
| submedial thalamus | **0** | 0 | 0 | **0.5** | 3 | 0 | **0.018** |
| periaqueductal gray pons | **0** | 0 | 0 | **0.5** | 4 | 0 | **0.018** |
| facial nucleus | **0** | 1 | 0 | **1.5** | 3 | 0 | **0.018** |
| pedunculopontine nucleus | **0** | 1 | 0 | **1.5** | 3 | 0 | **0.02** |
| dorsal medial hypothalamus | **0** | 1 | 0 | **1.5** | 4 | 0 | **0.02** |
| lateral amygdala | **2** | 5 | 0 | **5** | 10 | 1 | **0.021** |
| prelimbic ctx | **3** | 6 | 0 | **10** | 29 | 1 | **0.033** |
| accumbens shell | **0** | 2 | 0 | **6** | 16 | 0 | **0.034** |
| accumbens core | **0** | 2 | 0 | **1** | 8 | 0 | **0.037** |
| nucleus posterior commissure | **0** | 0 | 0 | **0** | 3 | 0 | **0.039** |
| paraventricular nuclus | **0** | 0 | 0 | **0** | 3 | 0 | **0.039** |
| ventral anterior-lateral thalamus | **0** | 1 | 0 | **1** | 14 | 0 | **0.048** |
| arcuate hypothalamus | **0** | 4 | 0 | **3** | 5 | 0 | **0.049** |
|  |  |  |  |  |  |  |  |
| substantia nigra compacta | **0** | 1 | 0 | **1** | 2 | 0 | **0.062** |

**Table 4.** ***Volume of Activation for Positive BOLD in TRPV1 Knockout and Wild-type Following Capsaicin***

Shown is a truncated list of 137 brain areas and their median (med, gray highlight), maximum (max) and minimum (min) number of voxels activated at 3-5 min post intradermal injection of capsaicin into the hindpaw of TRPV1 knockout and wild-type rats. The brain areas were rank ordered for their significance; the gray line delineates significant areas.
